# The causal relationship between immune cells and diabetic retinopathy: a Mendelian randomization study

**DOI:** 10.3389/fimmu.2024.1381002

**Published:** 2024-09-02

**Authors:** Yunyan Ye, Lei Dai, Hong Gu, Lan Yang, Zhangxing Xu, Zhiguo Li

**Affiliations:** ^1^ Department of Ophthalmology, Li Huili Hospital Affiliated with Ningbo University, Ningbo, China; ^2^ Department of Hepato-Pancreato-Biliary Surgery, Li Huili Hospital Affiliated with Ningbo University, Ningbo, China

**Keywords:** Mendelian randomization, immune cells, diabetic retinopathy, proliferative diabetic retinopathy, causal effect

## Abstract

**Purpose:**

This article explored the causal relationship between immune cells and diabetic retinopathy (DR) using single nucleotide polymorphisms (SNPs) as an instrumental variable and Mendelian randomization (MR).

**Methods:**

Statistical data were collected from a publicly available genome-wide association study (GWAS), and SNPs that were significantly associated with immune cells were used as instrumental variables (IVs). Inverse variance weighted (IVW) and MR−Egger regression were used for MR analysis. A sensitivity analysis was used to test the heterogeneity, horizontal pleiotropy, and stability of the results.

**Results:**

We investigated the causal relationship between 731 immune cells and DR risk. All the GWAS data were obtained from European populations and from men and women. The IVW analysis revealed that HLA DR on CD14+ CD16- monocytes, HLA DR on CD14+ monocytes, HLA DR on CD33-HLA DR+, HLA DR on CD33+ HLA DR+ CD14- on CD33+ HLA DR+ CD14dim, and HLA DR on myeloid dendritic cells may increase the risk of DR (P<0.05). HLA DR to CD14-CD16- cells, the monocytic myeloid-derived suppressor cell absolute count, the SSC-A count of CD4+ T cells, and terminally differentiated CD4+ T cells may be protective factors against DR (P<0.05). The sensitivity analysis indicated no heterogeneity or pleiotropy among the selected SNPs. Furthermore, gene annotation of the SNPs revealed significant associations with 10 genes related to the risk of developing PDR and potential connections with 12 other genes related to PDR.

**Conclusion:**

Monocytes and T cells may serve as new biomarkers or therapeutic targets, leading to the development of new treatment options for managing DR.

## 1 Background

Diabetic retinopathy (DR) is one of the most common microvascular complications of diabetes and affects 30% to 50% of diabetic patients. DR can progress to proliferative diabetic retinopathy (PDR) when the severity of ischemia increases, leading to neovascularization, fibroplasia, and retinal detachment, which are the leading causes of blindness and visual impairment in diabetic individuals. Diabetes is expected to affect 415 million people worldwide by 2024, more than one-third of whom suffer from DR, making it a serious global health issue (1, 2). Current DR treatment mostly focuses on regulating blood sugar, blood pressure, and lipid levels to slow down the disease and lower the risk of DR; however, there is still a high number of diabetes patients who develop PDR (3, 4). Early detection and diagnosis of DR, as well as systematic therapy, can prevent persistent vision loss; however, diagnosis and treatment of DR are often delayed due to a lack of resources for early DR screening (5). As a result, identifying more precise and sensitive biomarkers is critical for facilitating early detection of DR and understanding its pathophysiology (6).

Immune cells play a crucial role in the onset and progression of DR. In DR, there is frequent and persistent white blood cell adhesion to the vascular wall, which may result in capillary occlusion and retinal ischemia (7). They also play an important role in the pathogenesis of late PDR and can contribute to neovascularization, vitreous hemorrhage, and traction retinal detachment (8). A recent prospective study demonstrated that the number of circulating neutrophils increases while the number of T cells decreases during the initial stages and progression of DR (9). However, previous research on the pathophysiology of DR mostly relies on association analysis of observational cohorts, which cannot achieve causal association inference. Furthermore, the causal relationships between various immune cells and DR have not been investigated; therefore, there is limited existing evidence regarding immune cell types related to DR and their causal associations.

Mendelian randomization (MR) is a popular causal inference method in which the genetic variation associated with exposure is employed as an instrumental variable (IV) for assessing the causal effect of exposure on outcomes. It remains unaffected by common complicating variables such as acquired environment, life behavior, and habits, allowing it to minimize the reverse causal effect while maintaining maximum validity (10). Compared to traditional randomized controlled trials and observational research, MR can significantly reduce expenses and shorten study periods. It is widely employed in studies investigating the causal association of complex disorders, and the genome-wide association study (GWAS) dataset is expanding rapidly. These findings also provide a solid foundation for further MR research. With the advent of big data, the growth of epidemiological methodologies, and the demand for precision medicine, the application of MR for etiology mining will emerge as a new area of future research (11).

At present, no studies have been conducted to properly investigate the causal relationship between immune cells and DR using MR. Further investigation and study are required for diabetes. In this study, MR analysis was performed to investigate the causal relationship between immune cells and DR.

## 2 Methods

### 2.1 Study design

**Figure 1:**
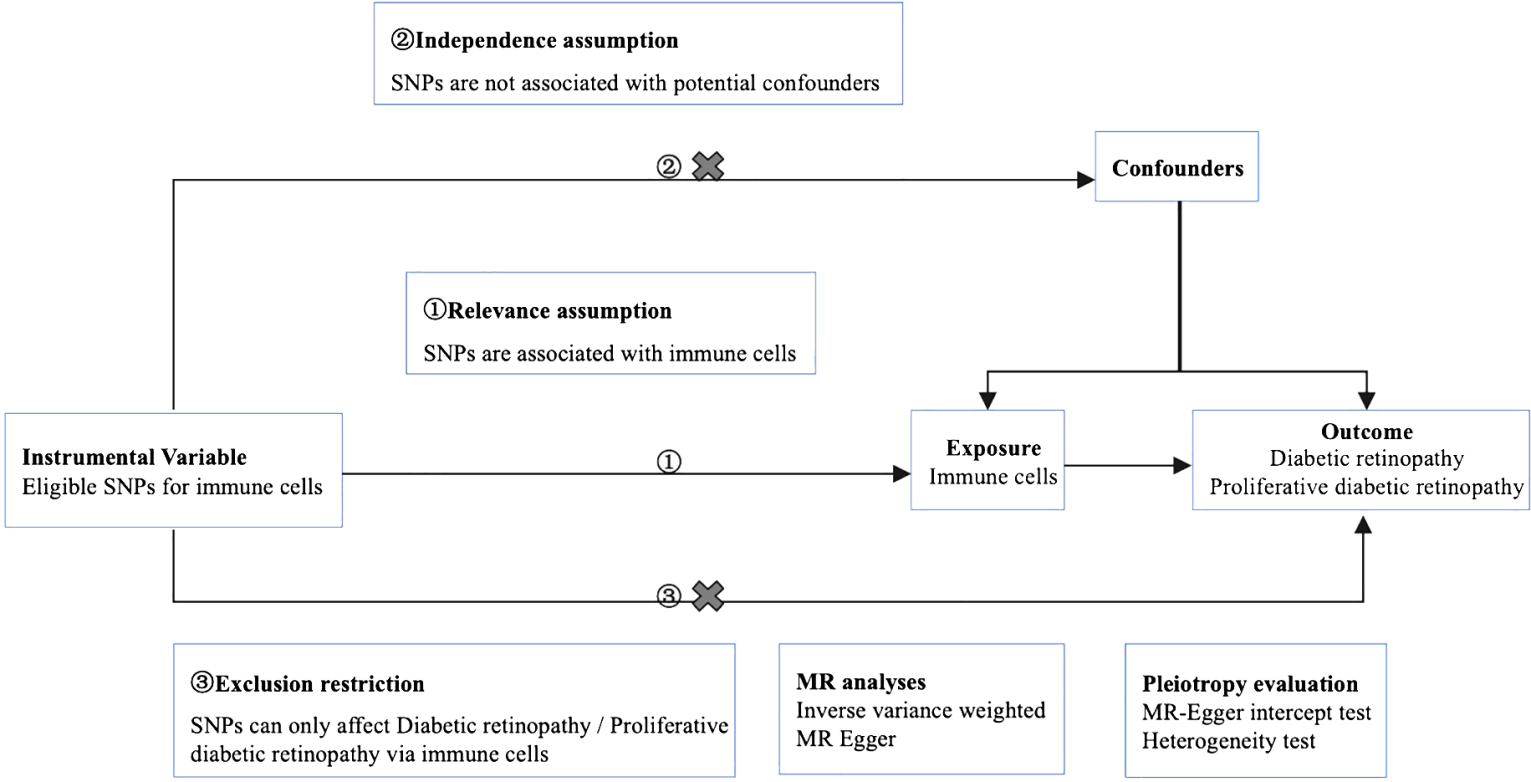
Mendelian randomization diagram of the association of immune cells with diabetic retinopathy. ① The genotype must be associated with the exposure to be studied. ② Genotypes must be independent of confounding factors. ③ Genotype is only associated with outcome by influencing the exposure factors to be studied.

We used MR analysis to evaluate the causal relationship between 731 immune cells and DR. In this study, immune cells were used as exposure factors and represented by X, whereas single nucleotide polymorphisms (SNPs) that were strongly linked with X were used as instrumental variables (IVs). The outcome variable was diabetic retinopathy. **Figure 1** depicts a schematic view of the study design, as well as the three essential MR assumptions (12).

### 2.2 Data source

The analysis was conducted using published summary statistics from the International Working Unit (IEU) Open GWAS project (https://gwas.mrcieu.ac.uk/), and it included 731 immune cells, two DR datasets (Finn-b-DM_RETINOPATHY and finn-b-H7_RETINOPATHYDIAB), and two PDR datasets (finn-b-DM_RETINA_PROLIF and finn-b-H7_RETINOPATHYDIAB_PROLIF). Validation was performed using the datasets finn-b-H7_RETINOPATHYDIAB and finn-b-H7_RETINOPATHYDIAB_PROLIF. The study was conducted on European individuals, including both men and women, and the summary data are provided in **Table 1**. The current analysis did not require ethics approval because all of the included GWASs received ethical review board approval and informed consent, as indicated in their individual original manuscripts.

**Table 1:** Detailed information of datasets.

Data source	Phenotype	Sample size	Cases	Population	Adjustment
IEU Open GWAS project	Immune cells	-	-	European	-
finn-b-DM_RETINOPATHY	Diabetic retinopathy (DM_RETINOPATHY)	-	14584	European	Males and Females
finn-b-H7_RETINOPATHYDIAB	Diabetic retinopathy (H7_RETINOPATHYDIAB)	-	3646	European	Males and Females
finn-b-DM_RETINA_PROLIF	Proliferative diabetic retinopathy (DM_RETINA_PROLIF)	-	8681	European	Males and Females
finn-b-H7_RETINOPATHYDIAB_PROLIF	Proliferative diabetic retinopathy (H7_RETINOPATHYDIAB_PROLIF)	-	1382	European	Males and Females

### 2.3 Selection and validation of SNPs

The selected SNPs were related to immune cells at a genome-wide significance threshold of *p* < 1×10^-5^. Second, pairwise linkage disequilibrium was used to assess the independence of the selected SNPs. When r^2^ > 0.001 (clumping window of 10,000 kb) was reached, the SNP that correlated with more SNPs or had a higher *P*-value was removed (**Figure 1**, ①). Phenoscanner was used to minimize the impact of improper SNPs (**Figures 1**, ②③). The *F*-statistic was subsequently used to validate the strength of each SNP. When the *F*-statistic exceeded 10, SNPs were deemed powerful enough to minimize the effects of potential bias. Furthermore, the SNPs listed above were retrieved from the GWAS summary data of DR and PDR, with a minimum r^2^ > 0.8. The information from the datasets listed above was summarized (12).

### 2.4 Mendelian randomization analysis

The causal association study was conducted using inverse variance weighting (IVW) and MR−Egger regression. The discrepancy in intercept terms, as indicated by the intercept of the MR-Egger analysis, revealed horizontal pleiotropy in the study. Cochrane’s Q value and accompanying *P*-values were used to assess heterogeneity among the selected IVs, with *P* > 0.05 indicating no heterogeneity. In addition, a leave-one-out (LOO) analysis was performed to observe whether a particular SNP had a disproportionate effect on the overall estimations. Forest plots were used to visualize the MR analysis results, while scatter plots and funnel plots were utilized to assess the stability of the MR data (13, 14).

The statistical power was calculated using an online tool at: http://cnsgenomics.com/shiny/mRnd/ (15). We used the following formula to calculate R^2^: (2×EAF×(1−EAF)×beta2)/[(2×EAF×(1−EAF)×beta2)+(2×EAF×(1−EAF)×N×SE(beta)2) (16).

### 2.5 SNP annotation

The SNPs were annotated using online tools (https://biit.cs.ut.ee/gprofiler/snpense). g: SNPense maps a collection of human SNP rs-codes to gene names, along with chromosome positions and expected variant effects. Mapping was allowed only for variations that coincided with at least one protein coding Ensembl gene. All underlying data were extracted from Ensembl variation data.

### 2.6 Statistical methods

All the statistical analyses were conducted using R 4.1.0 software and R packages. IVW and MR−Egger analyses were performed using the TwoSample MR package (α= 0.05), meta-analysis using the meta package, and a statistically significant difference was indicated by P < 0.05. If the null hypothesis was rejected, random effects IVW was utilized rather than fixed effects IVW (17). Additionally, the Forest Plats package was used to generate forest plots.

## 3 Results

### 3.1 Selected SNPs

A total of 6,196 SNPs in the DR and 6,186 in the PDR MR analyses were used, respectively (**Supplementary Table 2**). We obtained the degree of phenotype overlap from the FinnGen database. Among diabetic retinopathy phenotypes, there is a 57.47% sample overlap between the DM_RETINOPATHY cohort and the H7_retinydiab cohort. In terms of the proliferative diabetic retinopathy phenotype, there is a 25.82% sample overlap between the DM_RETINA_PROLIF cohort and the H7_retinyDIAB_prolif cohort (**Supplementary Table Overlap**).

### 3.2 MR analysis results

**Figure 2:**
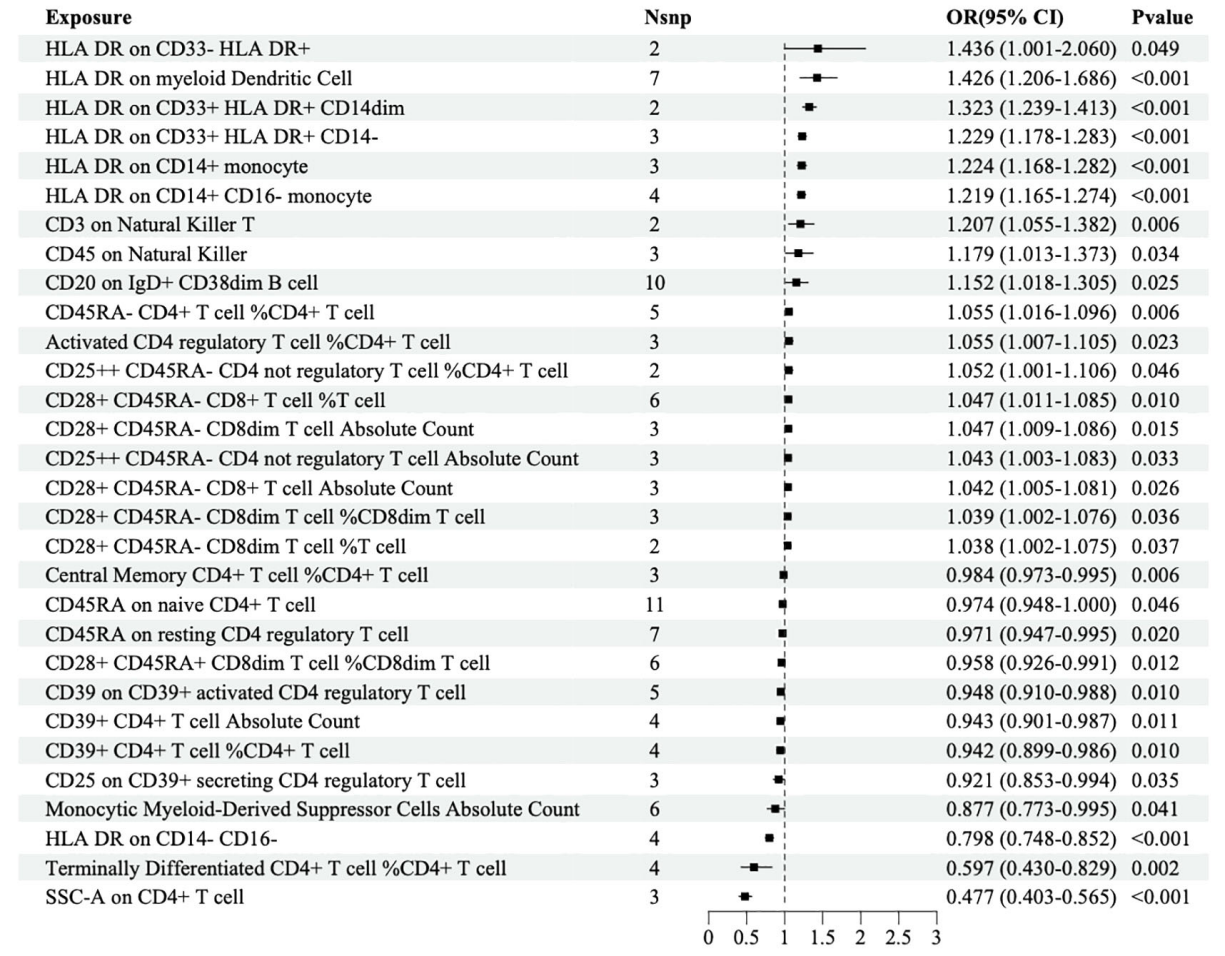
Forest map of MR causal effect between immune cells and DR (finn-b-DM_RETINOPATHY).

MR analysis was performed to explore the causal effects of immune cells on DR, and the IVW method was used as the primary analysis. According to MR analysis using the finn-b-DM_RETINOPATHY dataset, IVW analysis revealed that 30 immune cells were significantly associated with DR. In total, 18 immune cells were found to increase the risk of DR; for example, HLA DR was found in CD33+ HLA DR+ CD14- (OR=1.229, 95% CI=1.178-1.283, *P*<0.001), and HLA DR was found in CD33+ HLA DR+ CD14dim (OR=1.323, 95% CI=1.239-1.413, *P*<0.001). Furthermore, 12 immune cells, such as HLA-DR on CD14-CD16+ cells (odds ratio (OR)=0.798, 95% CI=0.748-0.852, *P*<0.001) and on CD4+ T cells (OR=0.477, 95% CI=0.403-0.565, *P*<0.001), may decrease the risk of DR (**Figure 2**).

**Figure 3:**
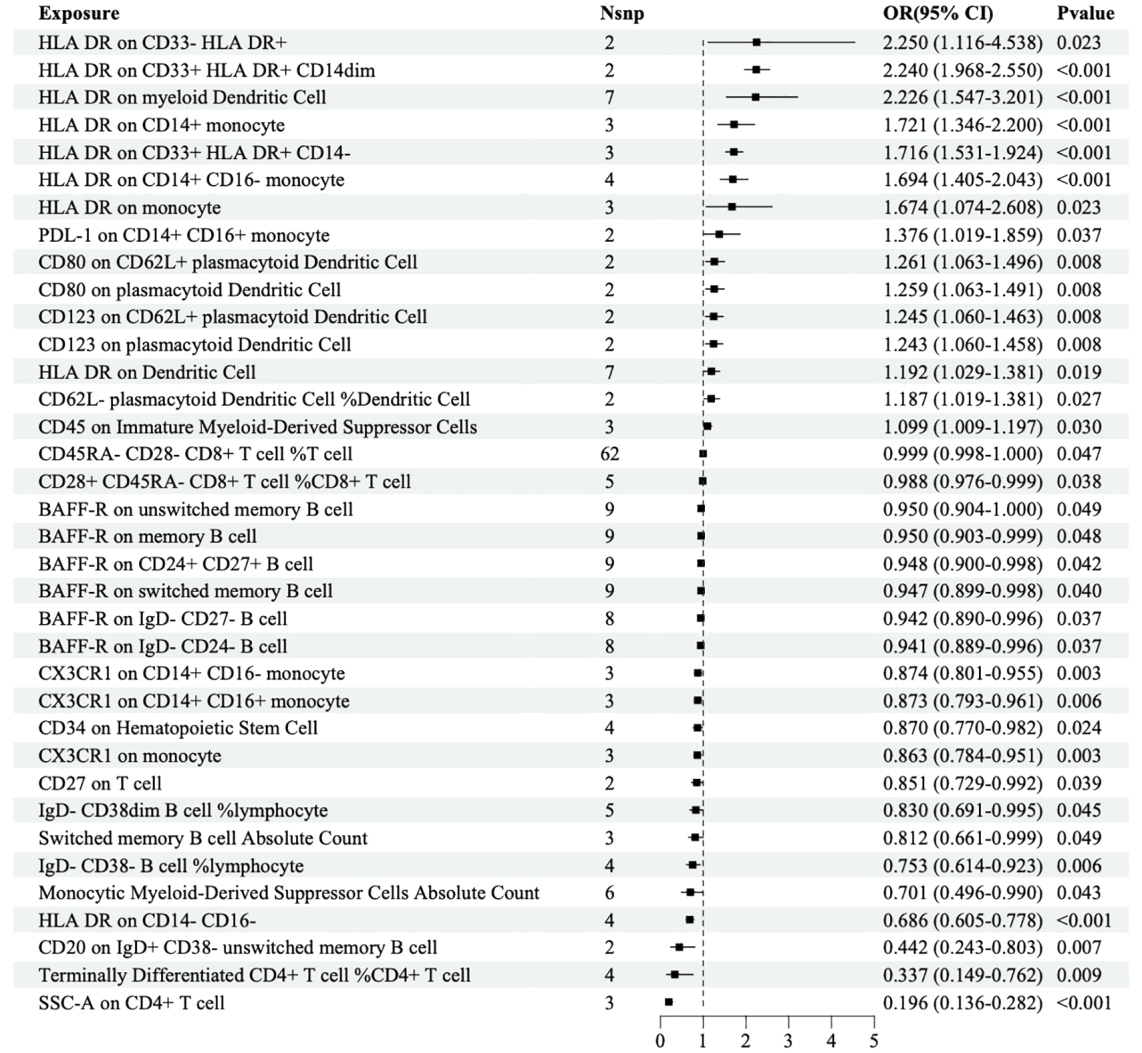
Forest map of MR causal effect between immune cells and DR (finn-b-H7_RETINOPATHYDIAB).

The IVW analysis based on the FinN-B-H7_RETINOPATHYDIAB dataset revealed that 36 immune cells were significantly associated with DR. Among these, HLA-DR among CD33+ HLA DR+ CD14- (OR=1.716, 95% CI=1.531-1.924, *P*<0.001) and HLA-DR among CD33+ HLA DR+ CD14dim (OR=2.240, 95% CI=1.968-2.550, *P*<0.001) were identified as two of the 15 immune cells that may increase the risk of DR. Additionally, HLA-DR among CD14- CD16- (OR=0.686, 95% CI=0.605-0.778, *P*<0.001) and SSC-A among CD4+ T cells (OR=0.196, 95% CI=0.136-0.282, *P*<0.001) were identified as two of the 21 immune cells that may decrease the risk of DR (**Figure 3**).

Merged MR analysis results from the FINN-B-H7_RETINYDIAB and FINN-b-DM_RETINOPATHY datasets revealed 10 immune cells. HLA-DR on myeloid dendritic cells, HLA-DR on CD14+ CD16- monocytes, HLA-DR on CD33+ HLA-DR+ CD14-, HLA-DR on CD14+ monocytes, HLA-DR on CD33- HLA-DR+, and HLA-DR on CD33+ HLA-DR+ CD14dim are six immune cells that may be risk factors for DR. Additionally, the following four immune cells may serve as protective factors for DR: monocytic myeloid-derived suppressor cell absolute count, terminally differentiated CD4+ T cell, HLA-DR on CD14- CD16-, and SSC-A on CD4+ T cells (**Table 2A**).

**Table 2A:** MR results of causal links.

Data source	Classification	Trait type	Panel	Nsnp	Methods	OR(95%CI)	P-value	FDR	Power
finn-b-DM_RETINOPATHY	HLA DR on myeloid Dendritic Cell	MFI	cDC	7	Inverse variance weighted	1.426(1.206-1.686)	3.32E-05	2.45E-03	1.000
	HLA DR on CD14+ CD16- monocyte	MFI	Monocyte	4	Inverse variance weighted	1.219(1.165-1.274)	4.41E-18	1.14E-15	1.000
	HLA DR on CD14- CD16-	MFI	Monocyte	4	Inverse variance weighted	0.798(0.748-0.852)	1.19E-11	1.02E-09	1.000
	HLA DR on CD14+ monocyte	MFI	Monocyte	3	Inverse variance weighted	1.224(1.168-1.282)	2.38E-17	3.07E-15	1.000
	SSC-A on CD4+ T cell	Morphological parameter	TBNK	3	Inverse variance weighted	0.477(0.403-0.565)	1.04E-17	1.78E-15	1.000
	HLA DR on CD33+ HLA DR+ CD14-	MFI	Myeloid cell	3	Inverse variance weighted	1.229(1.178-1.283)	1.91E-21	9.86E-19	1.000
	HLA DR on CD33+ HLA DR+ CD14dim	MFI	Myeloid cell	2	Inverse variance weighted	1.323(1.239-1.413)	8.11E-17	8.37E-15	1.000
finn-b-H7_RETINOPATHYDIAB	HLA DR on myeloid Dendritic Cell	MFI	cDC	7	Inverse variance weighted	2.226(1.547-3.201)	1.60E-05	1.18E-03	1.000
	HLA DR on CD14+ CD16- monocyte	MFI	Monocyte	4	Inverse variance weighted	1.694(1.405-2.043)	3.50E-08	3.61E-06	1.000
	HLA DR on CD14- CD16-	MFI	Monocyte	4	Inverse variance weighted	0.686(0.605-0.778)	4.49E-09	5.79E-07	1.000
	HLA DR on CD14+ monocyte	MFI	Monocyte	3	Inverse variance weighted	1.721(1.346-2.200)	1.47E-05	1.18E-03	1.000
	SSC-A on CD4+ T cell	Morphological parameter	TBNK	3	Inverse variance weighted	0.196(0.136-0.282)	1.63E-18	2.81E-16	1.000
	HLA DR on CD33+ HLA DR+ CD14-	MFI	Myeloid cell	3	Inverse variance weighted	1.716(1.531-1.924)	1.89E-20	4.86E-18	1.000
	HLA DR on CD33+ HLA DR+ CD14dim	MFI	Myeloid cell	2	Inverse variance weighted	2.240(1.968-2.550)	2.99E-34	1.54E-31	1.000

**Table 2B:** MR results of causal links.

Data source	Classification	Trait type	Panel	Nsnp	Methods	OR(95%CI)	P-value	FDR	Power
finn-b-DM_RETINA_PROLIF	HLA DR on myeloid Dendritic Cell	MFI	cDC	7	Inverse variance weighted	1.690(1.327-2.153)	2.15E-05	1.59E-03	1.000
	HLA DR on CD14+ CD16- monocyte	MFI	Monocyte	4	Inverse variance weighted	1.369(1.245-1.505)	8.20E-11	1.06E-08	1.000
	HLA DR on CD14+ monocyte	MFI	Monocyte	3	Inverse variance weighted	1.380(1.218-1.565)	4.59E-07	3.94E-05	1.000
	CD4 on CD39+ activated CD4 regulatory T cell	MFI	Treg	3	Inverse variance weighted	1.224(1.107-1.354)	8.53E-05	5.50E-03	0.994
	SSC-A on CD4+ T cell	Morphological parameter	TBNK	3	Inverse variance weighted	0.348(0.270-0.449)	3.78E-16	6.50E-14	1.000
	HLA DR on CD33+ HLA DR+ CD14-	MFI	Myeloid cell	3	Inverse variance weighted	1.383(1.305-1.466)	9.65E-28	3.62E-25	1.000
	HLA DR on CD33+ HLA DR+ CD14dim	MFI	Myeloid cell	2	Inverse variance weighted	1.599(1.469-1.740)	1.40E-27	3.62E-25	1.000
finn-b-H7_RETINOPATHYDIAB_PROLIF	HLA DR on myeloid Dendritic Cell	MFI	cDC	7	Inverse variance weighted	2.603(1.645-4.121)	4.46E-05	3.83E-03	1.000
	HLA DR on CD14+ CD16- monocyte	MFI	Monocyte	4	Inverse variance weighted	1.900(1.536-2.350)	3.27E-09	4.22E-07	1.000
	HLA DR on CD14+ monocyte	MFI	Monocyte	3	Inverse variance weighted	1.943(1.474-2.560)	2.40E-06	2.48E-04	1.000
	CD4 on CD39+ activated CD4 regulatory T cell	MFI	Treg	3	Inverse variance weighted	1.532(1.202-1.954)	5.73E-04	4.23E-02	0.992
	SSC-A on CD4+ T cell	Morphological parameter	TBNK	3	Inverse variance weighted	0.142(0.100-0.202)	8.73E-28	4.50E-25	1.000
	HLA DR on CD33+ HLA DR+ CD14-	MFI	Myeloid cell	3	Inverse variance weighted	1.879(1.525-2.315)	3.15E-09	4.22E-07	1.000
	HLA DR on CD33+ HLA DR+ CD14dim	MFI	Myeloid cell	2	Inverse variance weighted	2.654(2.162-3.257)	1.06E-20	2.73E-18	1.000

After merging the MR analysis results from the finn-b-DM_RETINA_PROLIF and finn-b-H7_RETINOPATHYDIAB_PROLIF datasets, 10 immune cell types were obtained. These include HLA DR on dendritic cells, HLA DR on myeloid dendritic cells, HLA DR on CD14+ CD16- monocytes, HLA DR on CD33+ HLA DR+ CD14- cells, CD4 on CD39+ activated CD4 regulatory T cells, HLA DR on CD14+ monocytes, HLA DR on CD33+ HLA DR+ CD14dim, and HLA DR on CD33- HLA DR+. These eight immune cell types may be risk factors for DR. The other two immune cell types may act as protective factors for PDR: HLA DR on CD14- CD16- and SSC-A on CD4+ T cells (**Table 2B**).

### 3.3 Sensitivity analysis

**Figure 4:**
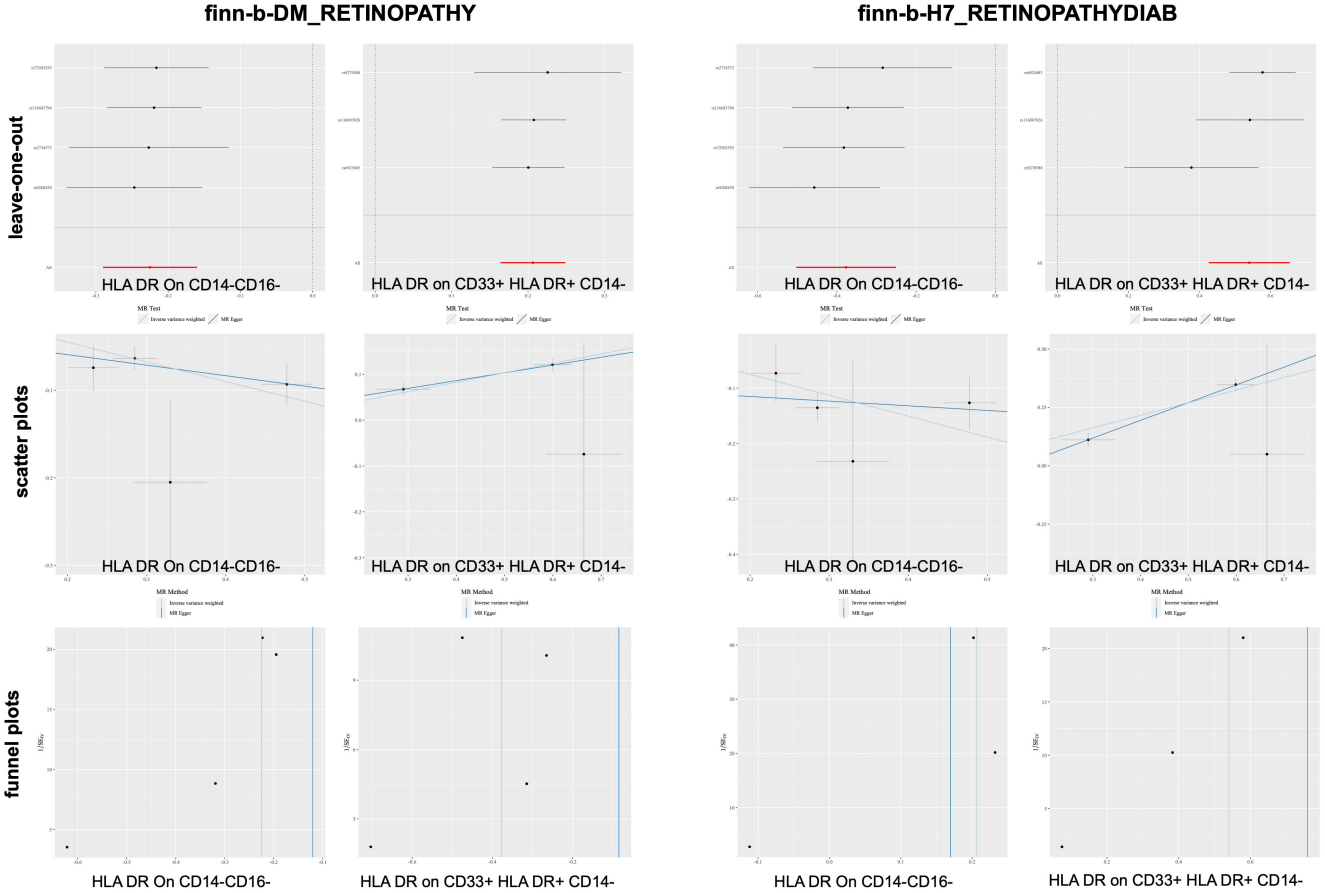
Sensitivity analysis for DR datasets.

According to the merging of the two DR datasets, Cochran’s Q *P*-value revealed no heterogeneity among SNPs in DR and immune cell HLA DR on CD14-CD16- or HLA DR on CD33+ HLA DR+ CD14- or DR (*P* > 0.05, **Table 3A**). Furthermore, the MR−Egger intercept ruled out the possibility of horizontal pleiotropy for these associations. The LOO sensitivity analysis revealed that no individual SNP disproportionately affected the overall estimates (**Figure 4**). Additionally, scatter plots and funnel plots also indicated the stability of the results (**Figure 4**).

**Table 3A:** Evaluation of heterogeneity and pleiotropy.

Data source	Classification	Trait type	Panel	Nsnp	Heterogeneity			Horizontal pleiotropy		
					I^2^(%)	Cochran’s Q	P-value	Egger intercept	SE	P-value
finn-b-DM_RETINOPATHY	HLA DR on myeloid Dendritic Cell	MFI	cDC	7	97	178.303	<0.001	-0.137	0.075	0.127
	HLA DR on CD14+ CD16- monocyte	MFI	Monocyte	4	0	1.611	0.657	-0.018	0.050	0.751
	HLA DR on CD14- CD16-	MFI	Monocyte	4	1	3.016	0.389	-0.035	0.046	0.521
	HLA DR on CD14+ monocyte	MFI	Monocyte	3	0	1.634	0.442	-0.052	0.066	0.574
	SSC-A on CD4+ T cell	Morphological parameter	TBNK	3	69	6.544	0.038	0.401	0.720	0.677
	HLA DR on CD33+ HLA DR+ CD14-	MFI	Myeloid cell	3	0	1.089	0.580	0.018	0.031	0.662
	HLA DR on CD33+ HLA DR+ CD14dim	MFI	Myeloid cell	2	0	0.164	0.685	-	-	-
finn-b-H7_RETINOPATHYDIAB	HLA DR on myeloid Dendritic Cell	MFI	cDC	7	97	218.238	<0.001	-0.288	0.166	0.144
	HLA DR on CD14+ CD16- monocyte	MFI	Monocyte	4	78	13.641	0.003	-0.297	0.145	0.177
	HLA DR on CD14- CD16-	MFI	Monocyte	4	0	2.821	0.420	-0.098	0.082	0.355
	HLA DR on CD14+ monocyte	MFI	Monocyte	3	86	14.335	<0.001	-0.476	0.128	0.168
	SSC-A on CD4+ T cell	Morphological parameter	TBNK	3	74	7.610	0.022	1.531	0.874	0.330
	HLA DR on CD33+ HLA DR+ CD14-	MFI	Myeloid cell	3	46	3.732	0.155	-0.109	0.061	0.325
	HLA DR on CD33+ HLA DR+ CD14dim	MFI	Myeloid cell	2	0	0.042	0.837	-	-	-

**Table 3B:** Evaluation of heterogeneity and pleiotropy.

Data source	Classification	Trait type	Panel	Nsnp	Heterogeneity			Horizontal pleiotropy		
					I^2^(%)	Cochran’s Q	P-value	Egger intercept	SE	P-value
finn-b-DM_RETINA_PROLIF	HLA DR on myeloid Dendritic Cell	MFI	cDC	7	97	228.471	<0.001	-0.198	0.109	0.128
	HLA DR on CD14+ CD16- monocyte	MFI	Monocyte	4	63	8.183	0.042	-0.143	0.080	0.217
	HLA DR on CD14+ monocyte	MFI	Monocyte	3	77	8.758	0.013	-0.241	0.084	0.213
	CD4 on CD39+ activated CD4 regulatory T cell	MFI	Treg	3	0	1.522	0.467	-0.223	0.289	0.583
	SSC-A on CD4+ T cell	Morphological parameter	TBNK	3	77	8.869	0.012	1.134	0.480	0.255
	HLA DR on CD33+ HLA DR+ CD14-	MFI	Myeloid cell	3	12	2.284	0.319	-0.032	0.051	0.647
	HLA DR on CD33+ HLA DR+ CD14dim	MFI	Myeloid cell	2	0	0.359	0.549	-	-	-
finn-b-H7_RETINOPATHYDIAB_PROLIF	HLA DR on myeloid Dendritic Cell	MFI	cDC	7	96	136.103	<0.001	-0.371	0.207	0.133
	HLA DR on CD14+ CD16- monocyte	MFI	Monocyte	4	57	7.015	0.071	-0.350	0.154	0.152
	HLA DR on CD14+ monocyte	MFI	Monocyte	3	72	7.234	0.027	-0.523	0.202	0.235
	CD4 on CD39+ activated CD4 regulatory T cell	MFI	Treg	3	0	0.174	0.917	0.265	0.696	0.769
	SSC-A on CD4+ T cell	Morphological parameter	TBNK	3	29	2.798	0.247	1.644	1.009	0.350
	HLA DR on CD33+ HLA DR+ CD14-	MFI	Myeloid cell	3	60	4.980	0.083	-0.204	0.096	0.281
	HLA DR on CD33+ HLA DR+ CD14dim	MFI	Myeloid cell	2	0	0.510	0.475	-	-	-

**Figure 5:**
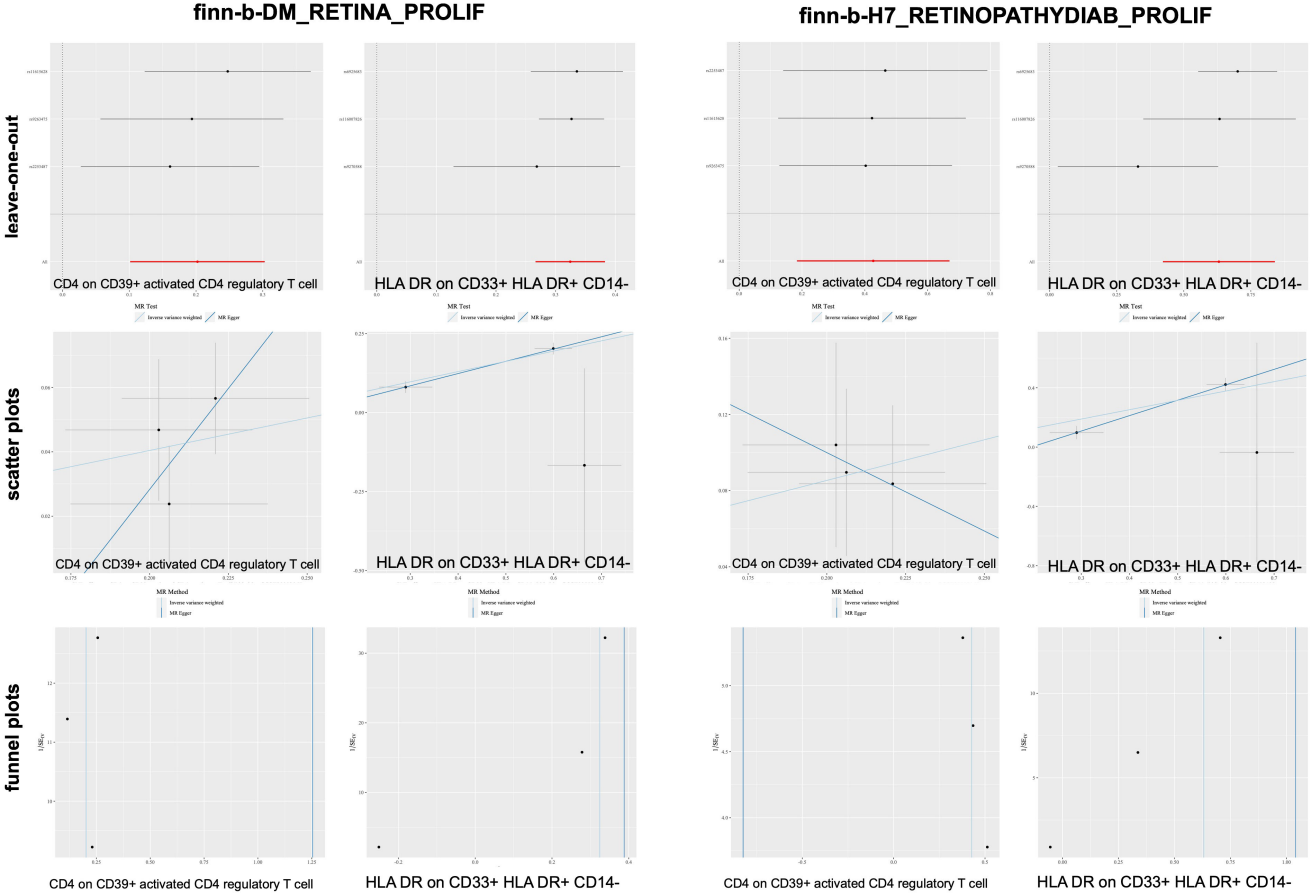
Sensitivity analysis for PDR datasets.

After merging the two PDR datasets, we detected no heterogeneity in the Cochran’s Q *P*- value among the SNPs of PDR and immune CD4+ T cells among the CD39+ activated CD4+ regulatory T cells or HLA DR among the CD33+ HLA DR+ CD14- cells (*P* > 0.05, **Table 3B**). Furthermore, the MR−Egger intercept ruled out the possibility of horizontal pleiotropy for these associations. The LOO sensitivity analysis revealed that no individual SNP disproportionately affected the overall estimates (**Figure 5**). In addition, the scatter plots and funnel plots also indicated the stability of the results (**Figure 5**).

In this study, we obtained seven immune cell features that were causally related to DR and PDR from two datasets. In the power calculations, the power of all the above immune cell features was >0.99, indicating that this study has sufficient statistical power (**Table 3**; **Supplementary Power-1**; **Supplementary Power-2**).

### 3.4 Meta-analysis

The MR data for immune cells from two DR patient datasets were merged through meta-analysis. If Cochran’s Q P value was <0.05, the random effects model was adopted. We identified six immune cells that have a risk effect on DR, namely, HLA DR on CD14+ CD16- monocytes, HLA DR on CD14+ monocytes, HLA DR on CD33- HLA DR+, HLA DR on CD33+ HLA DR+ CD14-, HLA DR on CD33+ HLA DR+ CD14dim, and HLA DR on myeloid dendritic cells. Additionally, we found four immune cells that have a protective effect against DR including HLA-DR on CD14- CD16-, monocytic myeloid-derived suppressor cell absolute count, and SSC-A on CD4+ T cells and terminally differentiated CD4+ T cells (**Supplementary DR-meta**).

The MR data for immune cells from two PDR patient datasets were merged through meta-analysis. We identified a risk effect of eight immune cells on PDR, including CD4+ on CD39+ activated CD4 regulatory T cells, HLA DR on CD14+ CD16- monocytes, HLA DR on CD14+ monocytes, HLA DR on CD33- HLA DR+, HLA DR on CD33+ HLA DR+ CD14-, HLA DR on CD33+ HLA DR+ CD14dim, HLA DR on dendritic cells, and HLA DR on myeloid dendritic cells. Additionally, we discovered the protective effects of two immune cell types on DR, namely, HLA DR on CD14- CD16- T cells and SSC-A on CD4+ T cells (**Supplementary PDR-meta**).

**Figure 6:**
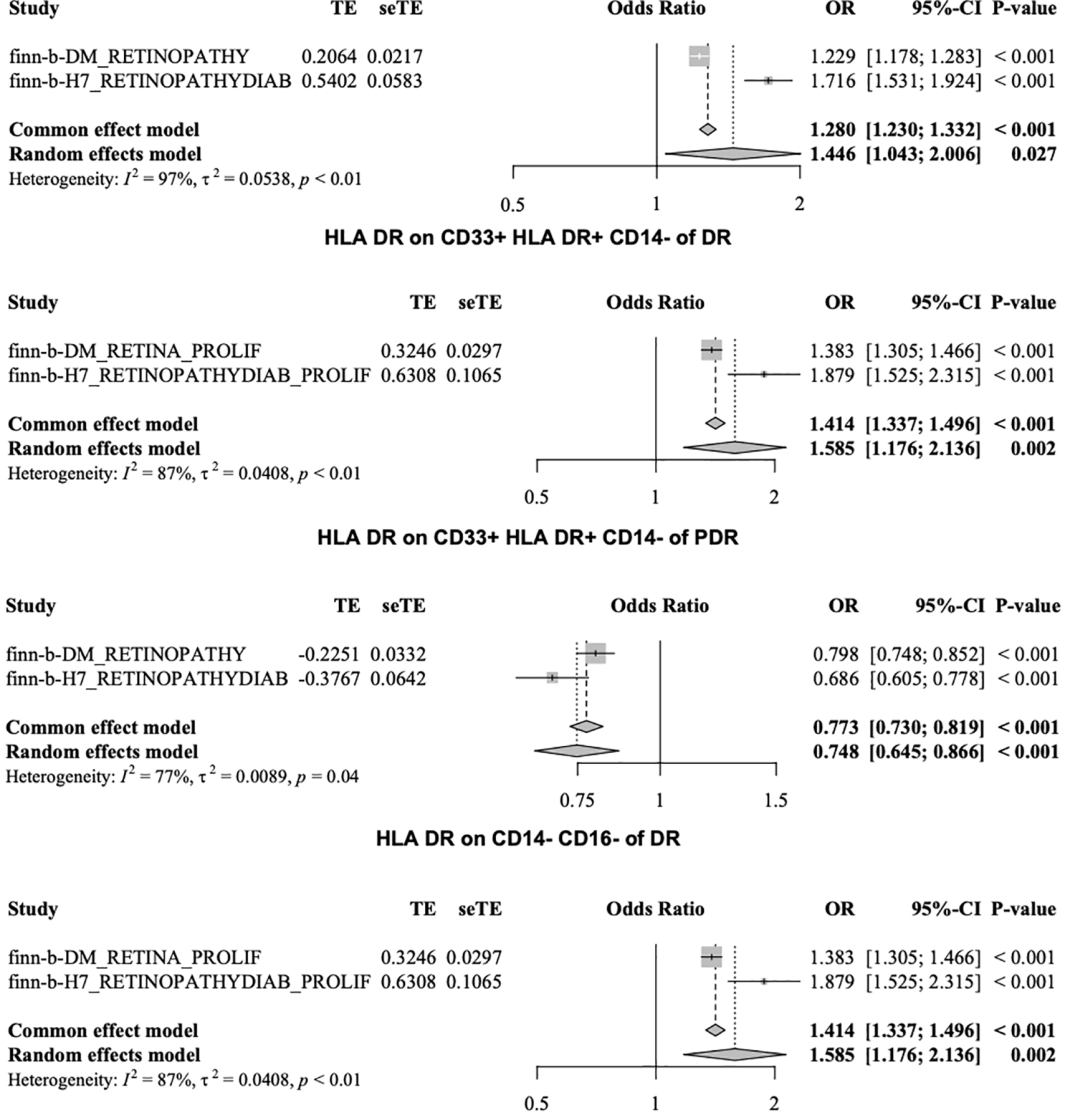
Results of meta-analysis.

The risk factors associated with the two phenotypes identified from the four datasets included six immune cell types: HLA-DR on CD14+ CD16 monocytes, HLA-DR on CD14+ monocytes, HLA-DR on CD33-HLA-DR+, HLA-DR on CD33+ HLA-DR+ CD14-, HLA-DR on CD33+ HLA-DR+ CD14dim, and HLA-DR on myeloid dendritic cells. Among the two phenotypes identified from the four datasets, HLA-DR to CD14-CD16- and SSC-A to CD4+ T cells were protective factors. **Figure 6** shows the HLA-DR on CD33+ cells, HLA-DR+CD14- cells, and HLA-DR on CD14-CD16- cells.

### 3.5 SNP annotation

Immune cell SNPs strongly associated with DR were annotated, and 10 genes potentially connected with PDR were identified. HLA-DPA1, CD33, HLA-DOB, and NEK7 may serve as protective factors for DR, while TSBP1-AS1, LYZ, ENSG00000233183, MICB, GABBR1, and FCGR3A may act as risk factors for DR (**Table 4A**).

**Table 4A:** SNP annotation.

Classification	Trait type	Panel	SNP	Chr	Start	End	Strand	Gene_ids	Gene_names
HLA DR on CD14- CD16-	MFI	Monocyte	rs116683794	6	33066337	33066337	+	ENSG00000231389	HLA-DPA1
HLA DR on CD14- CD16-	MFI	Monocyte	rs2734573		-1	-1			
HLA DR on CD14- CD16-	MFI	Monocyte	rs72502555		-1	-1			
HLA DR on CD14- CD16-	MFI	Monocyte	rs9268430	6	32377652	32377652	+	ENSG00000225914	TSBP1-AS1
HLA DR on CD14+ CD16- monocyte	MFI	Monocyte	rs150649461		-1	-1			
HLA DR on CD14+ CD16- monocyte	MFI	Monocyte	rs1800973	12	69350234	69350234	+	ENSG00000090382	LYZ
HLA DR on CD14+ CD16- monocyte	MFI	Monocyte	rs80032720		-1	-1			
HLA DR on CD14+ CD16- monocyte	MFI	Monocyte	rs9270585		-1	-1			
HLA DR on CD14+ monocyte	MFI	Monocyte	rs1800973	12	69350234	69350234	+	ENSG00000090382	LYZ
HLA DR on CD14+ monocyte	MFI	Monocyte	rs80032720		-1	-1			
HLA DR on CD14+ monocyte	MFI	Monocyte	rs9270585		-1	-1			
HLA DR on CD33+ HLA DR+ CD14-	MFI	Myeloid cell	rs116007826		-1	-1			
HLA DR on CD33+ HLA DR+ CD14-	MFI	Myeloid cell	rs6925683	6	33926515	33926515	+	ENSG00000233183	ENSG00000233183
HLA DR on CD33+ HLA DR+ CD14-	MFI	Myeloid cell	rs9270588		-1	-1			
HLA DR on CD33+ HLA DR+ CD14dim	MFI	Myeloid cell	rs142186496	6	31505930	31505930	+	ENSG00000204516	MICB
HLA DR on CD33+ HLA DR+ CD14dim	MFI	Myeloid cell	rs9270588		-1	-1			
HLA DR on myeloid dendritic cell	MFI	cDC	rs116007826		-1	-1			
HLA DR on myeloid dendritic cell	MFI	cDC	rs2858885		-1	-1			
HLA DR on myeloid dendritic cell	MFI	cDC	rs29221	6	29621347	29621347	+	ENSG00000204681	GABBR1
HLA DR on myeloid dendritic cell	MFI	cDC	rs35525122		-1	-1			
HLA DR on myeloid dendritic cell	MFI	cDC	rs55971447	1	1.62E+08	1.62E+08	+	ENSG00000203747,ENSG00000273112,ENSG00000289768	FCGR3A,ENSG00000273112,ENSG00000289768
HLA DR on myeloid dendritic cell	MFI	cDC	rs6925683	6	33926515	33926515	+	ENSG00000233183	ENSG00000233183
HLA DR on myeloid dendritic cell	MFI	cDC	rs9267650		-1	-1			
SSC-A on CD4+ T cell	Morphological parameter	TBNK	rs113243185		-1	-1			
SSC-A on CD4+ T cell	Morphological parameter	TBNK	rs148031710		-1	-1			
SSC-A on CD4+ T cell	Morphological parameter	TBNK	rs9271536		-1	-1			

“+” strand: sense strand, or coding strand.

“-” strand: antisense strand or template strand.

**Table 4B:** SNP annotation.

Classification	Trait type	Panel	SNP	Chr	Start	End	Strand	Gene_ids	Gene_names
CD4 on CD39+ activated CD4 regulatory T cell	MFI	Treg	rs11615628	12	6794465	6794465	+	ENSG00000010610	CD4
CD4 on CD39+ activated CD4 regulatory T cell	MFI	Treg	rs2253487	6	31281350	31281350	+	ENSG00000227939	RPL3P2
CD4 on CD39+ activated CD4 regulatory T cell	MFI	Treg	rs9263475		-1	-1			
HLA DR on CD14+ CD16- monocyte	MFI	Monocyte	rs150649461		-1	-1			
HLA DR on CD14+ CD16- monocyte	MFI	Monocyte	rs1800973	12	69350234	69350234	+	ENSG00000090382	LYZ
HLA DR on CD14+ CD16- monocyte	MFI	Monocyte	rs80032720		-1	-1			
HLA DR on CD14+ CD16- monocyte	MFI	Monocyte	rs9270585		-1	-1			
HLA DR on CD14+ monocyte	MFI	Monocyte	rs1800973	12	69350234	69350234	+	ENSG00000090382	LYZ
HLA DR on CD14+ monocyte	MFI	Monocyte	rs80032720		-1	-1			
HLA DR on CD14+ monocyte	MFI	Monocyte	rs9270585		-1	-1			
HLA DR on CD33+ HLA DR+ CD14-	MFI	Myeloid cell	rs116007826		-1	-1			
HLA DR on CD33+ HLA DR+ CD14-	MFI	Myeloid cell	rs6925683	6	33926515	33926515	+	ENSG00000233183	ENSG00000233183
HLA DR on CD33+ HLA DR+ CD14-	MFI	Myeloid cell	rs9270588		-1	-1			
HLA DR on CD33+ HLA DR+ CD14dim	MFI	Myeloid cell	rs142186496	6	31505930	31505930	+	ENSG00000204516	MICB
HLA DR on CD33+ HLA DR+ CD14dim	MFI	Myeloid cell	rs9270588		-1	-1			
HLA DR on myeloid dendritic cell	MFI	cDC	rs116007826		-1	-1			
HLA DR on myeloid dendritic cell	MFI	cDC	rs2858885		-1	-1			
HLA DR on myeloid dendritic cell	MFI	cDC	rs29221	6	29621347	29621347	+	ENSG00000204681	GABBR1
HLA DR on myeloid dendritic cell	MFI	cDC	rs35525122		-1	-1			
HLA DR on myeloid dendritic cell	MFI	cDC	rs55971447	1	1.62E+08	1.62E+08	+	ENSG00000203747,ENSG00000273112,ENSG00000289768	FCGR3A,ENSG00000273112,ENSG00000289768
HLA DR on myeloid dendritic cell	MFI	cDC	rs6925683	6	33926515	33926515	+	ENSG00000233183	ENSG00000233183
HLA DR on myeloid dendritic cell	MFI	cDC	rs9267650		-1	-1			
SSC-A on CD4+ T cell	Morphological parameter	TBNK	rs113243185		-1	-1			
SSC-A on CD4+ T cell	Morphological parameter	TBNK	rs148031710		-1	-1			
SSC-A on CD4+ T cell	Morphological parameter	TBNK	rs9271536		-1	-1			

“+” strand: sense strand, or coding strand.

Immune cell SNPs strongly associated with PDR were annotated, revealing 12 genes potentially related to PDR. CD4, RPL3P2, LYZ, TSBP1-AS1, ENSG00000233183, MICB, TVP23A, GABBR1, FCGR3A, and CIITA may be risk factors for PDR, however, HLA-DPA1 and TSBP1-AS1 might provide protection against it (**Table 4B**).

## 4 Discussion

A previous study revealed that immune system disorders and inflammation play important roles in the pathogenesis of DR. Further research into the specific role of immune mechanisms in DR, as well as the identification of more specific and sensitive biomarkers, will provide a new foundation and strategies for the early clinical diagnosis and treatment of DR (18). This MR study adds to the evidence supporting a causal connection between immune cells and DR. In DR, immune cells HLA DR on CD33+ HLA DR+ CD14- and HLA DR on CD33+ HLA DR+ CD14dim may be risk factors, while immune cells HLA DR on CD14-CD16- may be protective. In PDR, CD4+ T cells on CD39+ active CD4 regulatory T cells, HLA DR cells on CD14+ monocytes, and HLA DR cells on CD33+ HLA DR+ CD14dim may be risk factors. Immune cells, specifically those harboring SSC-A on CD4+ T cells, may provide protection. The findings of this study indicate that immune cells HLA DR on CD33+ HLA DR+ CD14- and HLA DR+ CD14- have a causative influence in both the datasets of DR and PDR, which may increase the risk of developing DR. Both types express molecules known as human leukocyte antigen (HLA) -driven receptors that are important markers for antigen-presenting cells (APCs). However, CD33+ cells are primarily present in monocytes or macrophages where they play a role in innate immunity and inflammation by exhibiting phagocytic activity along with cytokine production at sites experiencing inflammation. However, lymphocytes more widely express another type called CD14-, which has stronger associations with adaptive immune responses potentially contributing to diseases or immune response regulation.

Numerous studies have demonstrated that regulatory T cells and monocytes play a significant role in the pathogenesis of DR. The activation of immunoinflammatory cells and proinflammatory substances in the retinal tissue of DR patients contributes to the occurrence and progression of DR (19–22). Leukocyte adhesion stasis; neutrophil increase; abnormal expression of T cells, B lymphocytes, mononuclear/macrophages, and other immune cells; elevated concentrations of inflammatory and proangiogenic factors; and increased levels of anti-pericytes and anti-endothelial cell antibodies were found in the serum, vitreous, and retinal tissues of DR animal models and patients (23–25). YUAN et al. used a gene expression microarray for immunoinfiltration analysis. They found that in DR samples, there was significant overexpression (P<0.05) of seven types of immune cells: original B cells, plasma cells, memory CD4+ T cells, regulatory T cells (Tregs), MO macrophages, M1 macrophages, and neutrophils (*P*<0.05). The activated memory CD4+ T-cell module had the highest correlation and differential expression (*P*< 0.001). Activated NK cells showed low expression among immune cells (*P*<0.05) (26). Our study also revealed an increased risk for DR associated with HLA DR on CD14+ CD16- monocytes, HLA DR on CD33+ HLA DR+ CD14- cells, HLA DR on CD14+ monocytes, and HLA DR on CD33+ HLA DR+ CD14dim (all belonging to the monocyte population). Additionally, PDR patients showed a significant increase in CD4+ T cells on CD39+ activated CD4+ regulatory T cells. Two immune-associated target genes in DR, DLGAP5 and AURKB, were found to be enriched in pathways relevant to memory CD4+ cells. These findings suggest that DR is closely related to the activation of regulatory T cells and monocytes.

Our research revealed a significant increase in HLA-DR on CD33+HLA-DR+CD14- cells in both DR and PDR, indicating a strong correlation between microangiopathy in DR and the activation of myeloid-derived suppressor cells (MDSCs). MDSCs are diverse cell types that can effectively suppress T cell responses. Under normal conditions, these cells develop into dendritic cells, macrophages, and granulocytes. However, in pathological conditions such as infection, inflammation, or cancer, the differentiation of these cells stops resulting in their accumulation (27–29). Initially classified as HLA-DR-CD33+ or CD14-CD11b+ cells, both of which are populations of cells with T cell inhibitory activity (30, 31), human MDSCs can be further subdivided into granulocytic CD14− and monocytic CD14+ MDSCs (32, 33). One study found that patients with type 1 diabetes mellitus (T1D) have significantly greater numbers of MDSCs in their peripheral blood with M-MDSCs (CD14+ CD33+ HLA-DR−) being the most prevalent subset of MDSCs. Compared to diabetic patients without kidney disease, diabetic patients with kidney disease had a substantial increase in the number of total MDSCs and a rise in the percentage of CD14- cells (34). An imbalance of immune active cells is directly linked to the development of DR, as evidenced by the aberrant activation and expression of immune cells in the ocular tissue of DR patients and the association with DR. There are many similarities between diabetic nephropathy (DN) and DR. DN and DR are both microvascular complications resulting from diabetes, which are complex illnesses with diverse manifestations (35). If immune cell activation is effectively inhibited, delays in the onset of DR disease can be expected.

Our study indicates that CD33 is implicated in DR. Additionally, these findings reveal a set of genetic variants associated with proangiogenic and inflammatory pathways that may contribute to the pathogenesis of DR. Further investigation into these variants is necessary and may lead to the development of novel biomarkers and new therapeutic targets for DR. Previous research has shown that genes associated with angiogenesis and inflammatory pathways play a crucial role in the onset of DR (36–39). The Diabetic Retinopathy Genomics (DRGen) study revealed the involvement of Kruppel Like Factor 17 (KLF17), Zinc Finger Protein 395 (ZNF395), Myeloid cell surface antigen (CD33), Pleckstrin Homology Domain-Containing Family G Member 5 (PLEKHG5), NK2 Homeobox 3 (*NKX2.*3), and Collagen Type XVIII Alpha 1 Chain (COL18A1) in the progression of DR. These genes have been shown to be involved in angiogenesis and inflammatory pathways (40).

In MR studies, genetic variations that are substantially associated with an exposure are used as IVs to investigate the potential causal relationship between an exposure and a specific outcome of interest. Since genetic variants are randomly assigned at conception, MR estimates are not influenced by confounding factors, reverse causality, or measurement error (41). Inference typically relies on SNPs identified as IVs in GWASs. The current study was conducted in a rather conservative manner and supported by a comprehensive sensitivity analysis due to the strong assumptions underlying MR research (42). To ensure the robustness of the results, several measures were taken. Firstly, to minimize any bias resulting from demographic variability, only European populations were included in the analysis. Secondly, considering that both disease risk factors and immune cells are complex polygenic phenotypes that can be influenced by various genetic and environmental factors simultaneously (pleiotropy), we assessed potential pleiotropic effects through LOO and examined the intercept of MR-Egger regression. These approaches consistently yielded results suggesting reliable causal estimations. Data from European populations were utilized in this study to select a representative sample. By using MR methodology, it is possible to minimize the impact of reverse causation and confounding variables on estimation accuracy while producing trustworthy causal effect estimates based on observational research findings. Furthermore, GWAS data with large sample sizes were employed for these studies which significantly enhanced test efficiency compared to small-sample models relying on individual data points.

This study has certain limitations. First, there will inevitably be batch differences across the various datasets analyzed in this study due to its use of a public database. There are issues with the cohesiveness of integrating multiple databases in this study, and further efforts are needed to improve the accuracy of causal inference. Second, the research was limited to individuals with European ancestry, making it challenging to generalize the findings to other demographic groups. Third, residual and unmeasured confounders may still exist as the study was unable to determine whether demographic stratification and other potential confounders had an impact on its findings.

## 5 Conclusion

This study’s findings emphasized the complex network of connections between the immune system and DR, as it demonstrated causal relationships between various immune cells and DR through MR analysis. HLA-DR on CD14+ CD16 monocytes, HLA-DR on CD14+ monocytes, HLA-DR on CD33-HLA-DR+, HLA-DR on CD33+ HLA-DR+ CD14-, HLA-DR on CD33+ HLA-DR+ CD14dim, and HLA-DR on myeloid dendritic cells may increase the risk of DR. Additionally, HLA-DR to CD14-CD16- and SSC-A to CD4+ T cells may be protective factors against DR. These findings could open new avenues for investigating the biological causes of DR and pave the way for research into earlier intervention and treatment.

## Data Availability

The original contributions presented in the study are included in the article/**Supplementary Material**. Further inquiries can be directed to the corresponding author.
