# Supplementary figures and images for: The causal relationship between immune cells and diabetic retinopathy: a Mendelian randomization study
Source: Front Immunol. 2024 Sep 2;15:1381002. doi: 10.3389/fimmu.2024.1381002 (PMC11406504; doi:10.3389/fimmu.2024.1381002)

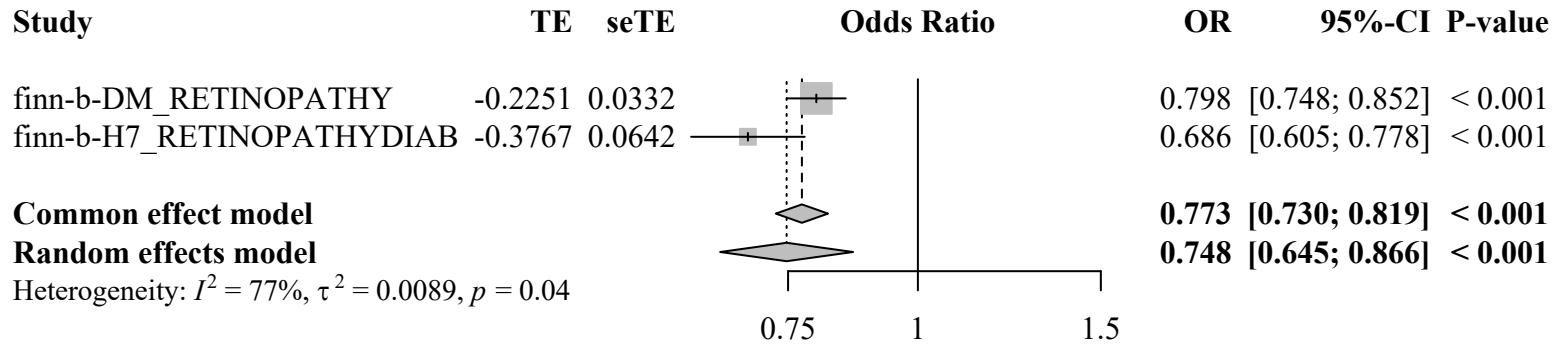

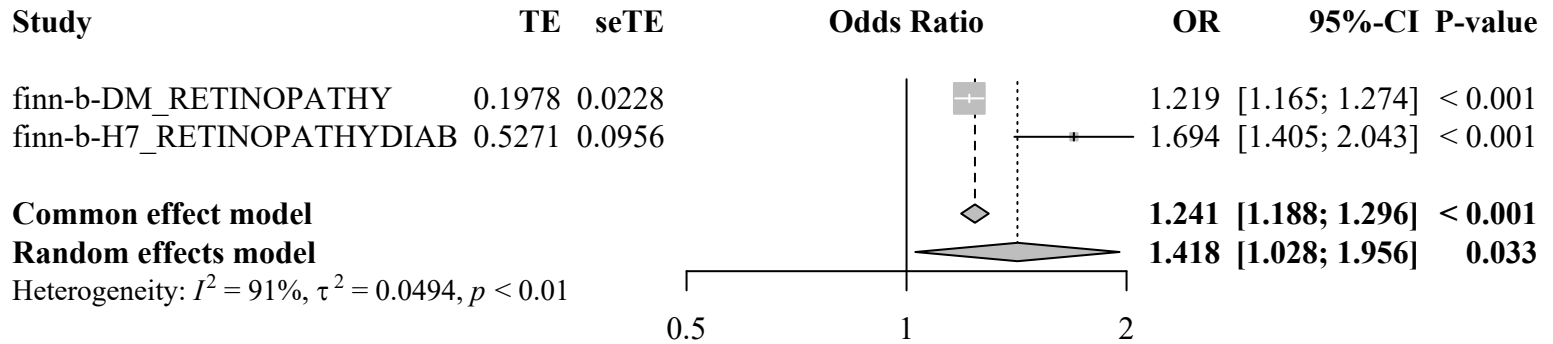

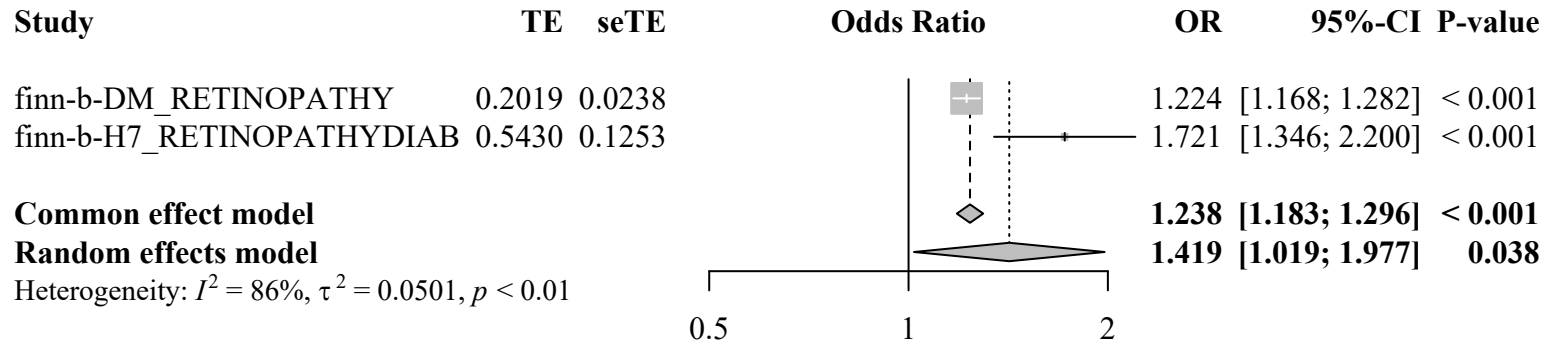

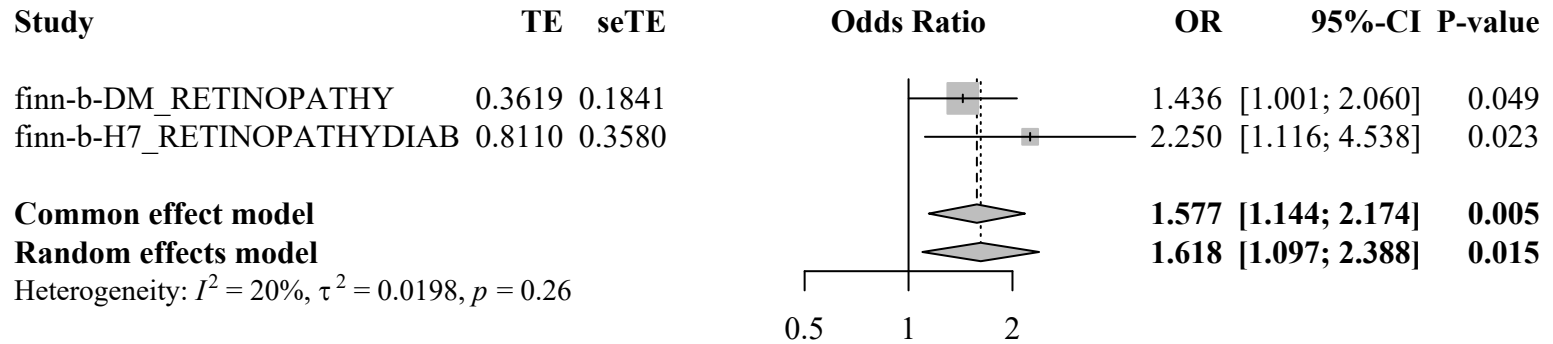

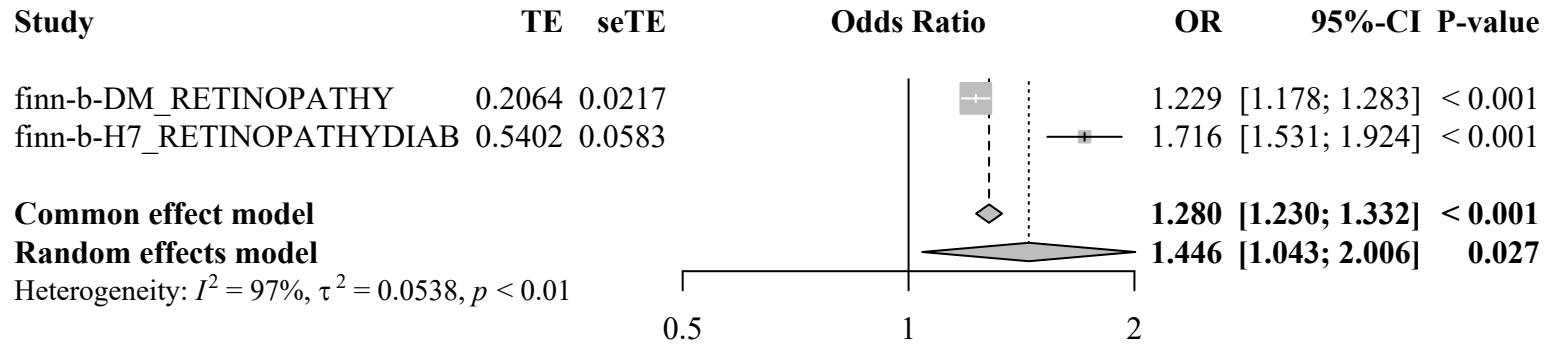

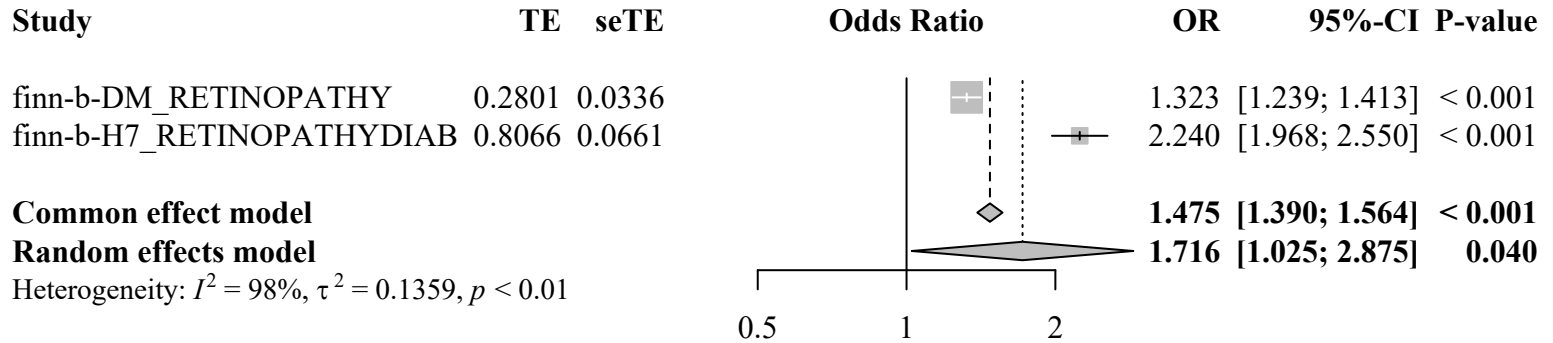

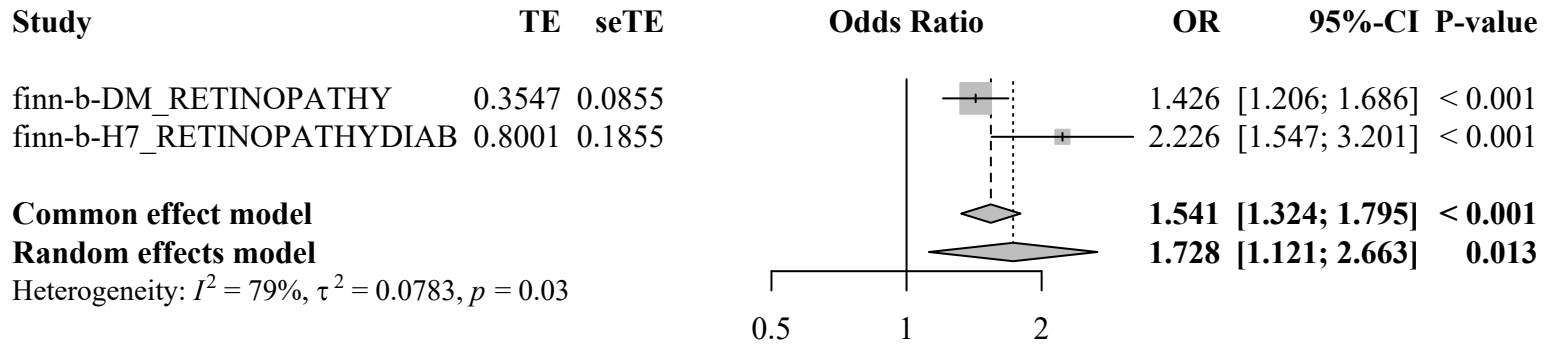

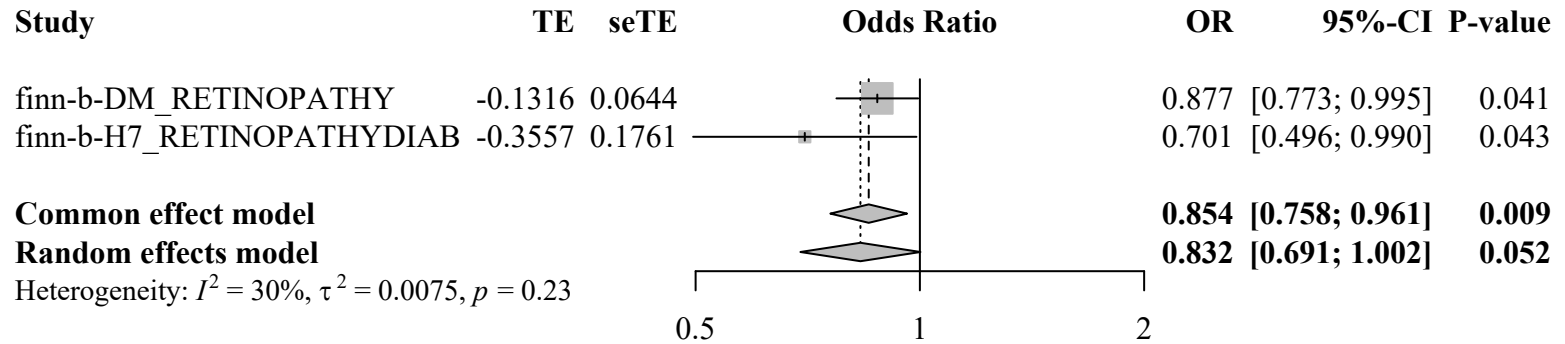

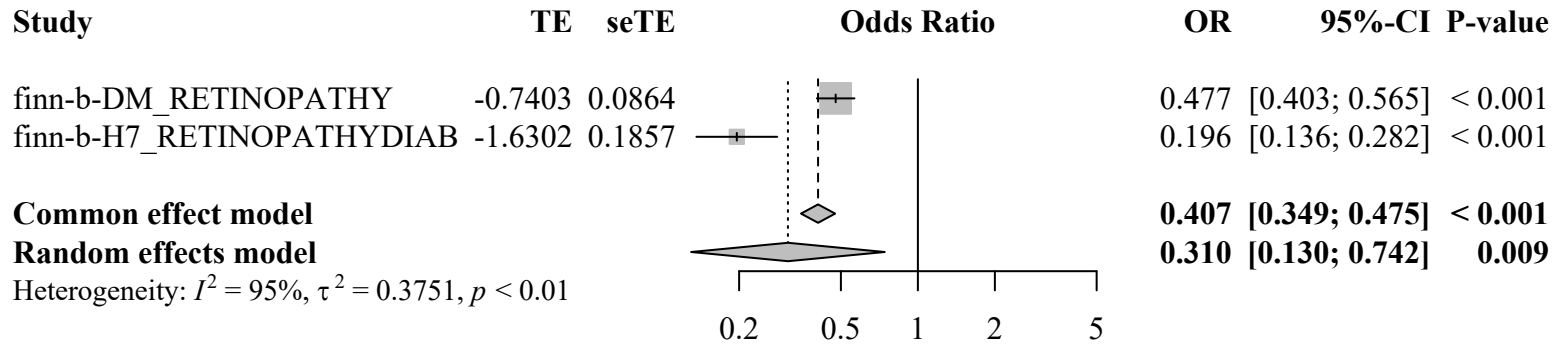

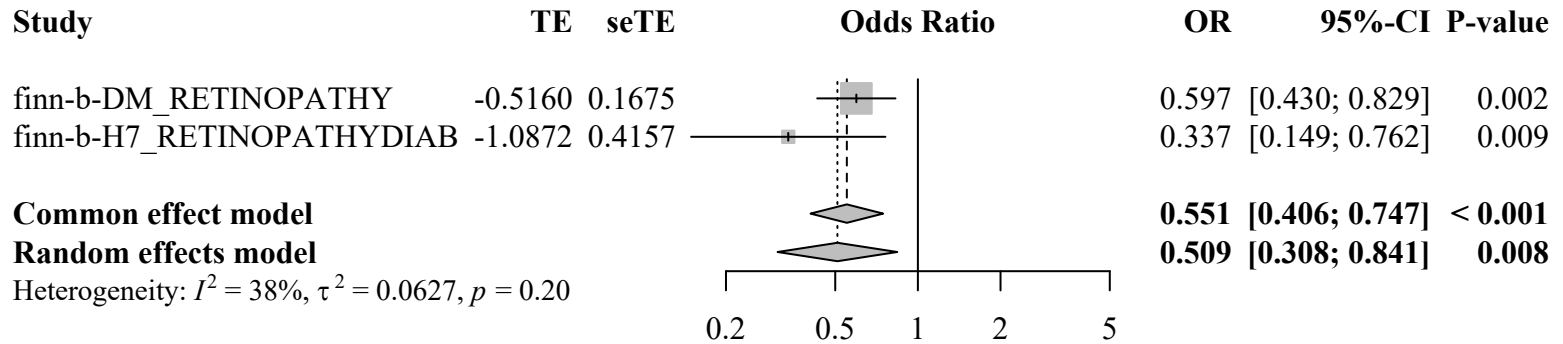

Supplement: Supplementary file 1 [file DataSheet1.pdf]
