# Supplementary material for: The causal relationship between immune cells and diabetic retinopathy: a Mendelian randomization study
Source: Front Immunol. 2024 Sep 2;15:1381002. doi: 10.3389/fimmu.2024.1381002 (PMC11406504; doi:10.3389/fimmu.2024.1381002)
Supplement: Supplementary file 4 [file DataSheet4.pdf]

Study	TE	seTE	Odds Ratio	OR	95%-CI	P-value
finn-b-DM_RETINA_PROLIF	0.2021	0.0514		1.224	[1.107; 1.354]	< 0.001
finn-b-H7_RETINOPATHYDIAB_PROLIF	0.4269	0.1240		1.532	[1.202; 1.954]	< 0.001
**Common effect model**				**1.265**	**[1.153; 1.389]**	**< 0.001**
**Random effects model**				**1.331**	**[1.076; 1.648]**	**0.009**

Heterogeneity: $I^2 = 64\%$, $\tau^2 = 0.0163$, $p = 0.09$

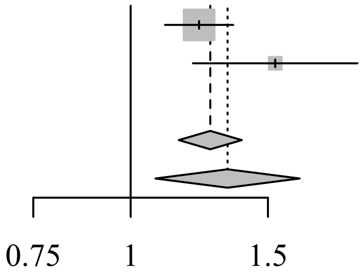

Study	TE	seTE	Odds Ratio	OR	95%-CI	P-value
finn-b-DM_RETINA_PROLIF	-0.2901	0.0563		0.748	[0.670; 0.836]	< 0.001
finn-b-H7_RETINOPATHYDIAB_PROLIF	-0.4552	0.1682		0.634	[0.456; 0.882]	0.007
**Common effect model**				**0.736**	**[0.663; 0.817]**	**< 0.001**
**Random effects model**				**0.736**	**[0.663; 0.817]**	**< 0.001**

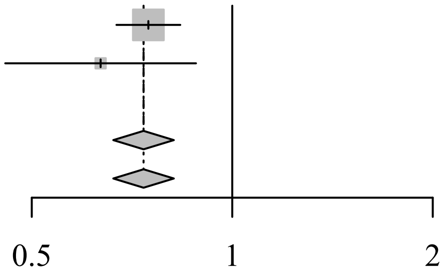

Heterogeneity: $I^2 = 0\%$, $\tau^2 = 0$, $p = 0.35$

Study	TE	seTE	Odds Ratio	OR	95%-CI	P-value
finn-b-DM_RETINA_PROLIF	0.3139	0.0483		1.369	[1.245; 1.505]	< 0.001
finn-b-H7_RETINOPATHYDIAB_PROLIF	0.6418	0.1085		1.900	[1.536; 2.350]	< 0.001
**Common effect model**				**1.445**	**[1.325; 1.576]**	**< 0.001**
**Random effects model**				**1.590**	**[1.154; 2.189]**	**0.005**

Heterogeneity: $I^2 = 87\%$, $\tau^2 = 0.0467$, $p < 0.01$

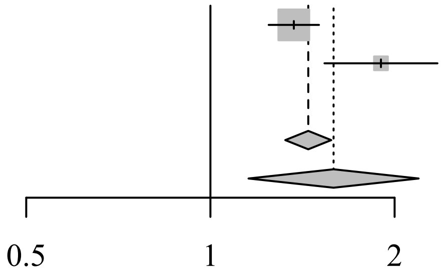

Study	TE	seTE	Odds Ratio	OR	95%-CI	P-value
finn-b-DM_RETINA_PROLIF	0.3223	0.0639		1.380	[1.218; 1.565]	< 0.001
finn-b-H7_RETINOPATHYDIAB_PROLIF	0.6640	0.1408		1.943	[1.474; 2.560]	< 0.001
**Common effect model**				**1.463**	**[1.306; 1.640]**	**< 0.001**
**Random effects model**				**1.600**	**[1.148; 2.230]**	**0.005**

Heterogeneity: $I^2 = 80\%$, $\tau^2 = 0.0464$, $p = 0.03$

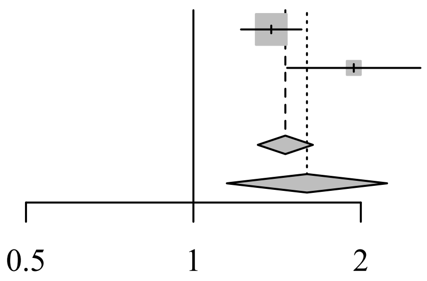

Study	TE	seTE	Odds Ratio	OR	95%-CI	P-value
finn-b-DM_RETINA_PROLIF	0.5344	0.2447		1.706	[1.056; 2.757]	0.029
finn-b-H7_RETINOPATHYDIAB_PROLIF	0.9981	0.3916		2.713	[1.259; 5.845]	0.011
**Common effect model**				**1.944**	**[1.294; 2.920]**	**0.001**
**Random effects model**				**1.945**	**[1.292; 2.929]**	**0.001**

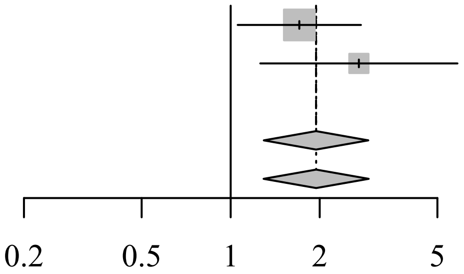

Heterogeneity: $I^2 = 1\%$, $\tau^2 = 0.0009$, $p = 0.32$

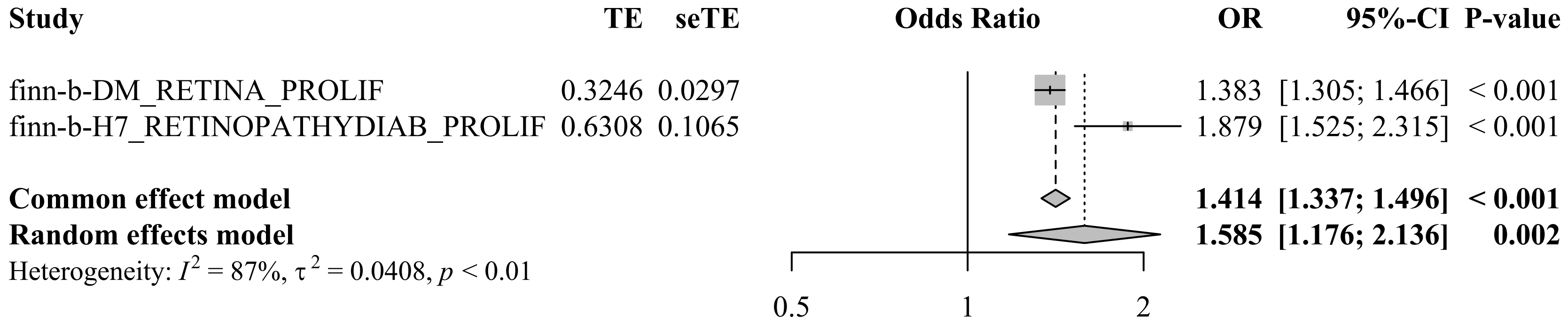
Study
TE
seTE
Odds Ratio
OR
95%-CI
P-value
finn-b-DM_RETINA_PROLIF 0.3246 0.0297 1.383 [1.305; 1.466] < 0.001
finn-b-H7_RETINOPATHYDIAB_PROLIF 0.6308 0.1065 1.879 [1.525; 2.315] < 0.001
Common effect model 1.414 [1.337; 1.496] < 0.001
Random effects model 1.585 [1.176; 2.136] 0.002
Heterogeneity: I^2 = 87%, τ^2 = 0.0408, p < 0.01
0.5
1
2

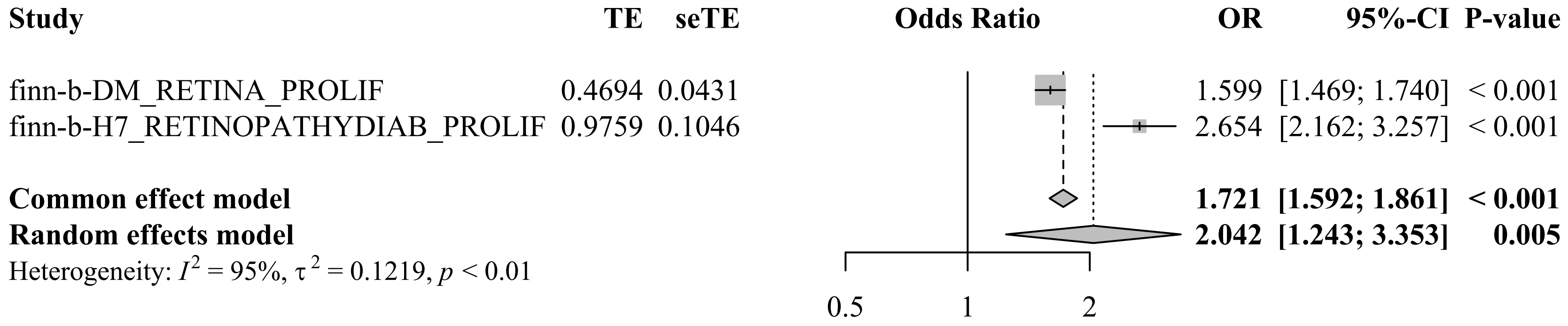
Study
TE
seTE
Odds Ratio
OR
95%-CI
P-value
finn-b-DM_RETINA_PROLIF 0.4694 0.0431 1.599 [1.469; 1.740] < 0.001
finn-b-H7_RETINOPATHYDIAB_PROLIF 0.9759 0.1046 2.654 [2.162; 3.257] < 0.001
Common effect model 1.721 [1.592; 1.861] < 0.001
Random effects model 2.042 [1.243; 3.353] 0.005
Heterogeneity: $I^2 = 95\%$, $\tau^2 = 0.1219$, $p < 0.01$
0.5
1
2

Study	TE	seTE	OR	95%-CI	P-value
finn-b-DM_RETINA_PROLIF	0.1021	0.0464	1.108	[1.011; 1.213]	0.028
finn-b-H7_RETINOPATHYDIAB_PROLIF	0.1537	0.0780	1.166	[1.001; 1.359]	0.049
**Common effect model**			**1.123**	**[1.038; 1.214]**	**0.004**
**Random effects model**			**1.123**	**[1.038; 1.214]**	**0.004**

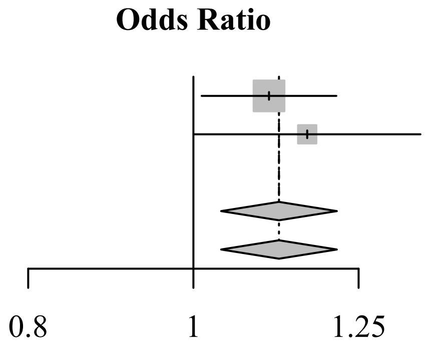

Heterogeneity: $I^2 = 0\%$, $\tau^2 = 0$, $p = 0.57$

Study	TE	seTE	Odds Ratio	OR	95%-CI	P-value
finn-b-DM_RETINA_PROLIF	0.5246	0.1235		1.690	[1.327; 2.153]	< 0.001
finn-b-H7_RETINOPATHYDIAB_PROLIF	0.9568	0.2344		2.603	[1.645; 4.121]	< 0.001
**Common effect model**				**1.856**	**[1.498; 2.299]**	**< 0.001**
**Random effects model**				**2.003**	**[1.324; 3.030]**	**0.001**

Heterogeneity: $I^2 = 62\%$, $\tau^2 = 0.0583$, $p = 0.10$

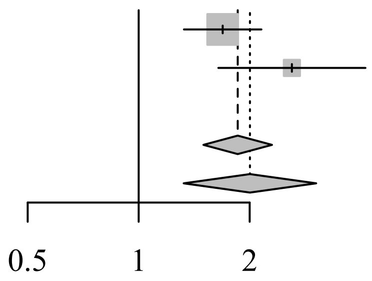

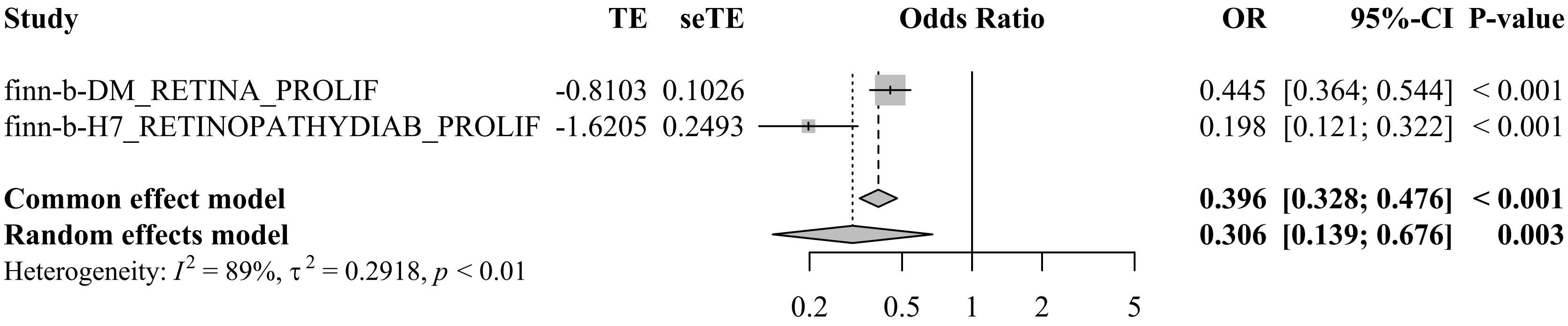
Study
TE
seTE
Odds Ratio
OR
95%-CI
P-value
finn-b-DM_RETINA_PROLIF -0.8103 0.1026 0.445 [0.364; 0.544] < 0.001
finn-b-H7_RETINOPATHYDIAB_PROLIF -1.6205 0.2493 0.198 [0.121; 0.322] < 0.001
Common effect model 0.396 [0.328; 0.476] < 0.001
Random effects model 0.306 [0.139; 0.676] 0.003
Heterogeneity: I^2 = 89%, τ^2 = 0.2918, p < 0.01
0.2
0.5
1
2
5
